# Numerical Investigation of Miniature Ejector Refrigeration System Embedded with a Capillary Pump Loop

**DOI:** 10.3390/mi8080235

**Published:** 2017-07-28

**Authors:** Jing-Ming Dong, He Song, Meng-Qi Yu, Wei-Ning Wang, Xin-Xiang Pan

**Affiliations:** Institute of Marine Engineering and Thermal Science, Marine Engineering College, Dalian Maritime University, Dalian 116026, China; yumengqi93@163.com (M.-Q.Y.); wn_wang@foxmail.com (W.-N.W.); panxx@dlmu.edu.cn(X.-X.P.)

**Keywords:** miniature steam ejector, CFD, area ratio, NXP, electronics cooling

## Abstract

A miniature steam ejector refrigeration system embedded with a capillary pump loop can result in a compact design which can be used for electronics cooling. In this paper, computational fluid dynamics (CFD) is employed to investigate the effects of the area ratio of the ejector constant-area mixing section to the nozzle throat, the length of the constant-area section, and the nozzle exit position (NXP), on the performance of a miniature steam ejector. Results show that the performance of the miniature steam ejector is very sensitive to the area ratio of the constant-area mixing section to the nozzle. For the needs of practical application, the area ratio of the constant-area mixing section to the nozzle should be smaller than 16 when the temperature of the primary flow is 60 °C. The NXP plays an important role in the flow phenomena inside the miniature ejector. The critical back pressure is more sensitive to length of the constant-area mixing section than the entrainment ratio. Results of this investigation provided a good solution to the miniature steam ejector embedded with a capillary pump loop for electronics cooling application.

## 1. Introduction

**Figure 1:**
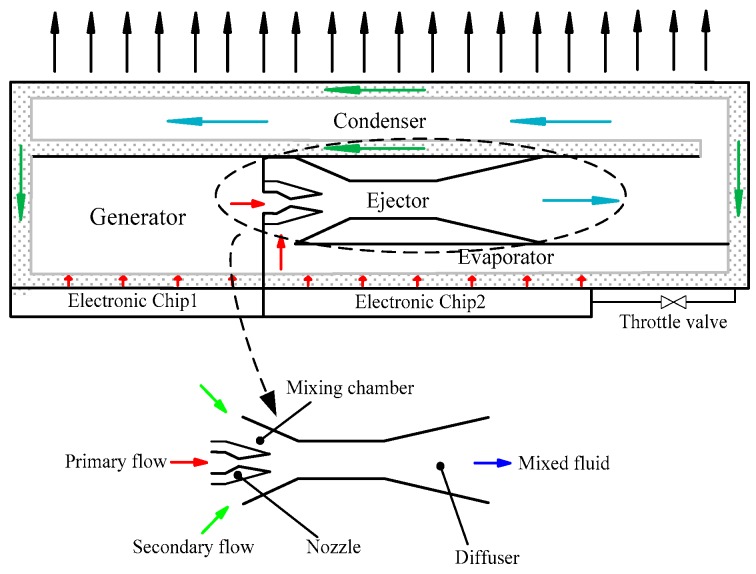
The schematic of the heat pipe/ejector system.

With the continuous integration of electronic components, the increasing heat flux means that heat dissipation in electronics is becoming more difficult to deal with [1]. Due to its excellent heat transfer characteristics, heat pipe is applied in electronic cooling [2]. However, it does not maintain the temperature of the electronics below the ambient temperature. Thermoelectric cooling, which consumes enormous amounts of electricity, is given great attention as an applicable method for electronic cooling [3,4]. In order to reduce power consumption, it is possible to use the heat dissipation of electronics to drive cooling plants. Riffat [5] proposed a novel concept of refrigeration, which is called the heat pipe/ejector cooling system. It is based on the combination of a heat pipe and an ejector. The schematic of the heat pipe/ejector system is shown in Figure 1. The system consists of ejector, condenser, capillary pump, generator, evaporator, and throttle valve. The capillary pump is employed to replace the electric circulation pump in the conventional ejector refrigeration system. The generator is powered by the heat dissipation of electronic devices. The vapor generated in the generator is called primary flow. The primary flow accelerates in the nozzle of the ejector and leaves the nozzle exit with supersonic speed, which creates a very low pressure in the mixing chamber. Subsequently, the secondary flow is entrained into the mixing chamber due to the pressure difference between the mixing chamber and the evaporator. In the mixing chamber and the constant-area mixing section, the primary flow and the secondary flow mix with each other. During the mixing process, shock waves appear and the velocity drops dramatically. The pressure increases when mixed fluid flows into the diffuser. After exiting the ejector, the mixed fluid enters into the condenser and is condensed into liquid. Some of the liquid returns to the evaporator through the throttle valve. The other part of the liquid comes back to the generator through the capillary pump. In the years since then, Smirnov [6] and Shi [7,8,9,10] investigated the feasibility and influence factors of this concept from different perspectives, such as the wick [10], structure of the ejector [9] and working fluids [7]. According to the first and second law of thermodynamics, the basic characteristics of the heat pipe/ejector system, such as entrainment ratio, thermal efficiency and exergy efficiency of the system, were investigated [11]. After that, the research on this concept has almost stalled.

Since the development of CFD technology, it has been widely used in the study of ejectors. Meanwhile, CFD is a useful approach to investigate the flow behavior in ejectors. Yang [12] investigated the flow phenomena inside steam ejectors numerically. The simulation results showed characteristics of the mixing process clearly. The entrainment ratio depended on the mixing degree of the primary and secondary flow in the mixing area, but the increase of the entrainment ratio might induce high mechanical energy loss and critical back pressure decrease. CFD was utilized to investigate the effects of operating parameters and geometries on the performance of ejectors [13]. Moreover, the phenomena on mixing behavior, choke flow, jet core effect and the shock waves have also been studied [14]. Ruangtrakoon [15] investigated the influence of the nozzle geometries on the performance of an ejector used in the steam jet refrigeration system. The results indicated that the CFD technique can be utilized to efficiently predict the performance of a steam ejector. Varga [16] studied performance of ejectors with variable primary nozzle geometry numerically. The research showed that the entrainment ratio is very sensitive to variations in operating conditions and a moveable spindle in the primary nozzle can improve the performance of the ejectors. Yan [17] employed a CFD model to estimate the effects of six geometry parameters on the entrainment ratio of an air-cooled ejector cooling system, two of the most important geometries that were most sensitive to the performance of the ejector were the area ratio of the constant-area mixing section to the nozzle and NXP, and the length of the constant-area mixing section, which affected the performance slightly. Varga [18] investigated the influence of the area ratio of the constant-area mixing section to the nozzle, NXP and constant-area mixing section length on the performance of an ejector using water as the working fluid. Both the area ratio of the constant-area mixing section to the nozzle and NXP greatly impacted the entrainment ratio and the critical back pressure, while the constant-area mixing section length had a little effect on the entrainment ratio. Pianthong [19] employed CFD to predict performance the of steam ejectors using water as the refrigerant (T_g_ = 120 °C–140 °C and T_e_ = 5 °C–15 °C); it was found that CFD could predict ejector characteristics very well. The investigation provided information on optimizing the ejector performance. Varga [20] first estimated the different ejector efficiencies by means of theoretical computing and CFD analysis. The research also described that the entrainment ratio increased with the increase of the area ratio. Zhu [21] investigated converging angle of mixing section and NXP by CFD. The investigation concluded that the ejector performance improved as the nozzle moved away from the mixing section. It was suggested that the converging angle of mixing section should be enlarged when the primary flow pressure increased. Wu [22] employed the CFD technique to study the effects of the mixing chamber geometries on the entrainment performance of steam ejectors. The study found that there is an optimum range for the convergence angle and chamber length where the ejector could obtain the maximal entrainment ratio.

On the basis of previous research, the miniature ejector refrigeration system embedded with a capillary pump has many predictable advantages. But the investigation on the performance of the miniature ejector has rarely been reported. In order to promote the application of the miniature ejector in electronic cooling, in this paper CFD is used to investigate the effects of the area ratio of ejector constant-area mixing section to nozzle throat, the length of constant-area section, and the nozzle exit position (NXP) on the performance of the miniature ejector and the flow phenomena inside the miniature ejector.

## 2. CFD Modeling

**Figure 2:**
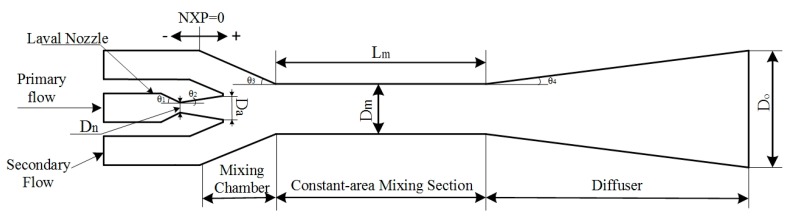
Geometry of the ejector.

The ejector is designed based on the constant pressure mixing model, which is more flexible to operate in wider ranges of condensing pressure [19]. Water was selected as the working fluid in the system. The latent heat of water is higher than most of other refrigerants. Also, water is a kind of environmentally friendly refrigerant. The model used in the CFD simulation comprises four parts: the nozzle, the mixing chamber, the constant-area mixing section and the diffuser. The effects of the area ratio of the constant-area mixing section to the nozzle (γ_A_), the length of constant-area mixing section ($L_{m}$) and the nozzle exit position (NXP) on the performance of the miniature ejector are investigated in this study. The geometry of the ejector is shown in Figure 2. The diameters of the constant-area mixing section are 6 mm, 7 mm and 8 mm. The length of the constant-area mixing section changes from 5 mm to 45 mm with 5 mm intervals. The NXP is defined as zero when the nozzle exit is located at the mixing chamber inlet. The nozzle exit position changes from −5 mm to 18 mm. The remaining parameters are fixed values. The specific geometrical parameters of the ejector are listed in Table 1.

In this paper, commercial software ICEM CFD 14.5 (ANSYS, Canonsburg, PA, USA) is used to generate a 2D grid. In order to accelerate the calculation and save time, the ejector geometry is set as axisymmetric in the ICEM CFD. The grid elements were initially created in the form of a quadrangular of about 80,000 elements. Later, the grid elements were increased to about 100,000 elements. This was to ensure that the simulated results were independent from the number of grid elements. The mesh quality is higher than 0.95 for each geometry. In addition, Fluent 14.5 is used as the CFD solver. The axisymmetric model is good enough to give accurate results [19]. The axis-symmetric swirl is applied in this paper.

For further numerical investigation, the following assumptions are made:

(a) The flow inside the ejector is in steady state.
(b) The primary flow at the ejector inlet is dry saturated vapor.
(c) Vapor expansion in the nozzle is isentropic.
(d) The secondary flow at the ejector inlet is dry saturated vapor.
(e) The inner wall of the ejector is adiabatic.
(f) Water vapor is assumed as ideal gas.

Based on the assumptions, the governing equations can be written as follows:

The continuity equation:

(1) $$\frac{\partial \rho }{\partial t}+\frac{\partial }{\partial x_{i}}\left({\rho u}_{i}\right)=0,$$

The momentum equation:

(2) $$\frac{\partial }{\partial t}\left({\rho u}_{i}\right)+\frac{\partial }{\partial x_{j}}\left({\rho u}_{i}u_{j}\right)=-\frac{\partial P}{\partial x_{i}}+\frac{\partial \tau_{\mathrm{ij}}}{\partial x_{j}},$$

The energy equation:

(3) $$\frac{\partial }{\partial t}\left(\rho E\right)+\frac{\partial }{\partial x_{i}}\left[u_{i}\left(\rho E+P\right)\right]=\vec{\nabla }\cdot \left(\alpha_{\mathrm{eff}}\frac{\partial T}{\partial x_{i}}\right)+\vec{\nabla }\cdot \left[u_{j}\left(\tau_{\mathrm{ij}}\right)\right],$$

(4) $$\rho =\frac{p}{\mathrm{RT}}$$

with $\tau_{\mathrm{ij}}=\mu_{\mathrm{eff}}\left(\frac{\partial u_{i}}{\partial x_{j}}+\frac{\partial u_{j}}{\partial x_{i}}\right)-\frac{2}{3}\mu_{\mathrm{eff}}\frac{\partial u_{k}}{\partial x_{k}}\delta_{\mathrm{ij}}$

And the k − ε model equations:

(5) $$\frac{\partial }{\partial t}\left(\rho k\right)+\frac{\partial }{\partial x_{i}}\left({\rho \mathrm{ku}}_{i}\right)=\frac{\partial }{\partial x_{j}}\left[\left(\mu +\frac{\mu_{t}}{\delta_{k}}\right)\frac{\partial k}{\partial x_{j}}\right]+G_{k}+G_{b}-\rho \varepsilon -Y_{M},$$

(6) $$\frac{\partial \left(\rho \varepsilon \right)}{\partial t}+\frac{\partial \left({\rho \varepsilon u}_{i}\right)}{\partial x_{i}}=\frac{\partial }{\partial x_{j}}\left[\left(\mu +\frac{\mu_{t}}{\delta_{\varepsilon }}\right)\frac{\partial \varepsilon }{\partial x_{j}}\right]+{\rho C}_{1}S\varepsilon -{\rho C}_{2}\frac{\varepsilon^{2}}{k+\sqrt{v\varepsilon }}+C_{1\varepsilon }\frac{\varepsilon }{k}C_{3\varepsilon }G_{b},$$

with $C_{1}=\max\left(\left[0.43,\frac{\mu }{\eta +5}\right]\right),\;\eta =S\frac{k}{\varepsilon }\;S=\sqrt{2S_{\mathrm{ij}}S_{\mathrm{ij}}}$ $S_{\mathrm{ij}}=\frac{1}{2}\left[\frac{\partial u_{i}}{\partial x_{j}}+\frac{\partial u_{j}}{\partial x_{i}}\right]$

In these equations, $C_{1}=1.44$ $C_{3}=0.09$ $C_{2}=1.9$ $\delta_{k}=1.0$ and $\delta_{\varepsilon }=1.3$

The realizable k − ε model can both predict the spreading rate of both planar and curved jets accurately and provide superior performance for flows involving boundary layers under strong adverse pressure gradients and separation [15]. In addition, the realizable k − ε turbulence model is recommended to solve the high velocity flow problem [12,13,14,19,22,23,24]. Thus, the viscous model is set as realizable k − ε turbulence model in this simulation. The “*standard wall functions*” are selected to treat the near wall treatment. The second-order upwind scheme is employed to discretize the convective terms. The “*pressure inlet*” condition is adopted on both the primary and secondary inlets flow boundary conditions. Besides, the ejector outlet is set as “*pressure outlet*” condition. For each simulation, the solution was iterated until the scaled residual for each governing equation was less than 10^−5^.

## 3. Results and Discussion

In an ejector refrigeration system, the design of the ejector has a great impact on the system’s performance. There are two common standards to evaluate the performance of an ejector, one is the entrainment ratio and another is known as the critical back pressure. More precisely, ω is defined as the entrainment ratio:

(7) $$\omega =\frac{\mathrm{mass}\;\mathrm{flow}\;\mathrm{of}\;\mathrm{the}\;\sec\mathrm{ond}\;\mathrm{fluid}}{\mathrm{mass}\;\mathrm{flow}\;\mathrm{of}\;\mathrm{the}\;\mathrm{primary}\;\mathrm{fluid}}=\frac{m_{e}}{m_{g}}$$

**Figure 3:**
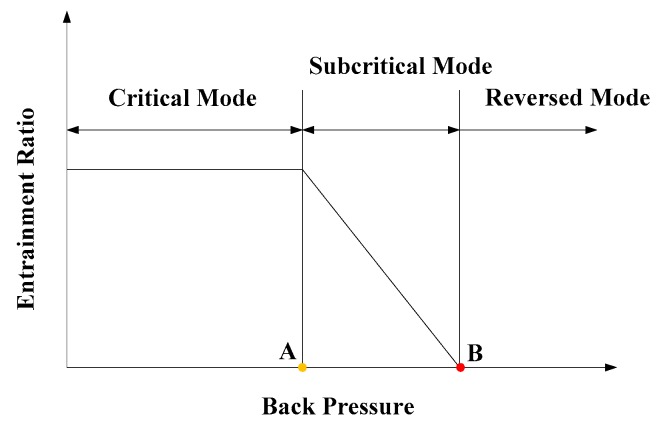
Performance characteristic of an ejector.

Figure 3 is the performance characteristic of an ejector. The characteristic curve can be divided into three modes [12]: critical mode, subcritical mode and reversed mode. The pressure at point A is named as the critical back pressure and the pressure at point B is called the break down pressure. Normally, the ejector works at the critical mode, and the entrainment ratio keeps unchanged with increasing back pressure. After the back pressure exceeds the critical back pressure, the entrainment ratio drops linearly with increasing back pressure. When the back pressure is higher than the back down pressure, the ejector cannot function and backflow may happen in the reversed mode region.

In order to investigate the performance of the miniature steam ejector applied in electronic cooling, a series of simulations have been conducted. The temperature of primary flow (T_g_) is 60 °C, and the temperature of secondary flow (T_e_) is 10 °C. The performance of the miniature steam ejector with a certain geometrical construction was investigated by changing the temperature of the condenser (T_c_). The results are summarized and discussed as follows.

### 3.1. Effect of Area Ratio of Constant-Area Mixing Section to the Nozzle Throat

**Figure 4:**
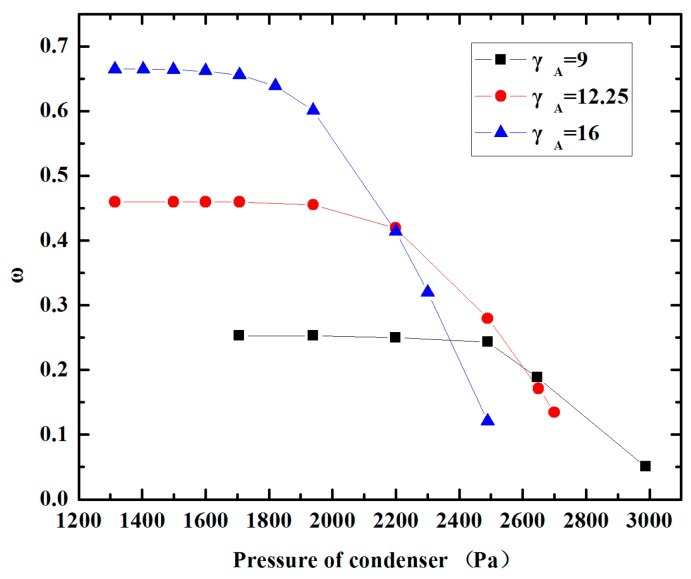
Variation of performance with different area ratios.

The area ratio of the constant-area mixing section to the nozzle throat is the most important parameter affecting the performance of the ejector. In this section, NXP equals 8 mm, $L_{m}$ is fixed as 30 mm. By changing the area ratio of the constant-area mixing section to the nozzle throat (γ_A_), the effects of the area ratio on the entrainment ratio and the critical back pressure of the miniature ejector are investigated. The γ_A_ is set as 9, 12.25 and 16. The results of CFD simulations are shown in Figure 4. It can be seen clearly that the entrainment ratio ω increases sharply when γ_A_ increases. It is shown that the entrainment ratios are about 0.26, 0.46, 0.66 for γ_A_ = 9, 12.25 and 16, respectively. They have a difference of about 0.2. Besides, the data of the simulation results reveal that the critical back pressure drops dramatically. Correspondingly, the critical back pressure for γ_A_ = 9, 12.25 and 16 are 2480 Pa, 2142 Pa and 1761 Pa. It can be concluded that the entrainment ratio and critical back pressure are both sensitive to γ_A_. So the area ratio of the constant-area mixing section to the nozzle throat should be designed carefully.

**Figure 5:**
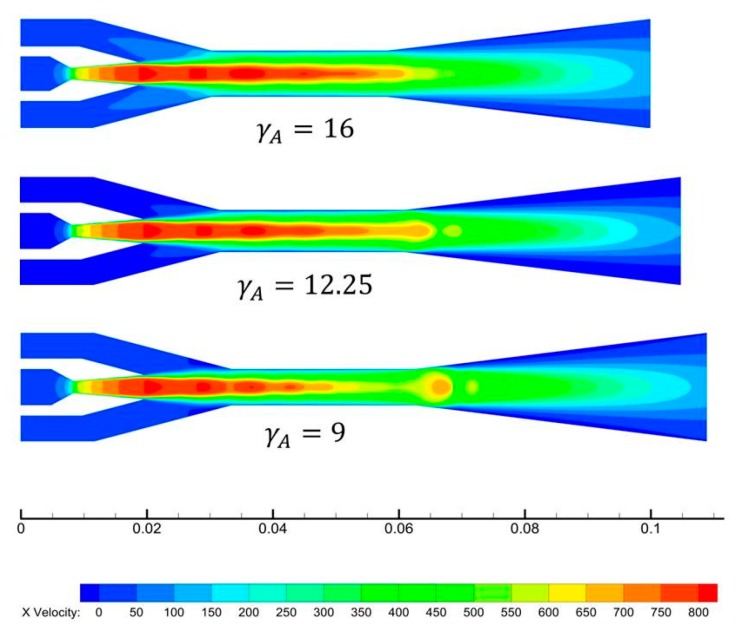
Velocity contours at different area ratios.

The velocity contours of ejectors with different γ_A_ are shown in Figure 5 for further study. It can be seen that the primary flow accelerates from subsonic speed to supersonic speed in the Laval nozzle, and the velocity along the axis increases continuously after exiting the nozzle. A series of normal shock waves called the shock train can be observed downstream of the nozzle exit section. According to the law of momentum conservation, a higher primary flow velocity is corresponding to a smaller primary flow pressure. The pressure of the primary flow drops to about 1000 Pa at the outlet of the nozzle. As a result, the secondary flow is entrained into the mixing chamber spontaneously at T_e_ = 10 °C.

**Figure 6:**
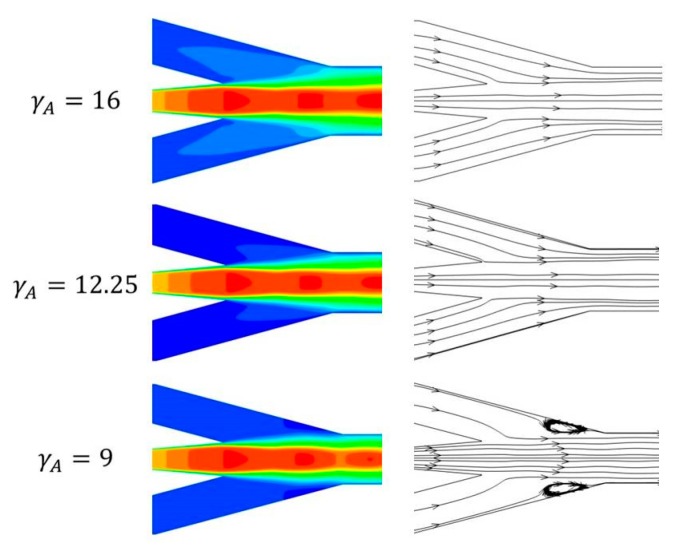
Localized patterns of ejectors.

Figure 6 is the localized velocity contours and stream trace line in mixing chamber. From Figure 6, a ‘*light-colored wing-shaped*’ area can be seen clearly when γ_A_ = 16. Then, the pressure in this area is lower than other places in the mixing chamber, and the ‘*light-colored wing-shaped*’ area becomes smaller with decreasing γ_A_. In other words, the low pressure area in the mixing chamber becomes larger when γ_A_ increases. Therefore, the entrainment ratio increases. Focus on the detail views of the stream trace line pattern in Figure 6, there is a small vortex around the outlet end of the mixing chamber when γ_A_ = 9. However, the vortex disappeared when γ_A_ = 12.25 and 16. The reason is that when γ_A_ is larger than 9, the primary flow expands adequately in the mixing chamber and the pressure of the primary flow can drop to lower level, hence the entrainment ratio can reach a higher value. However, increasing the area ratio improves the entrainment ratio, but also induces a low critical back pressure. From the point of energy loss, a larger area ratio gives more space for the mixing flow to expand and mix, resulting in the energy loss of the mixing flow along the radial direction increases correspondingly. This results in reduced the critical back pressure. In conclusion, for a given working condition, decreasing the γ_A_ resulted in a worse entrainment ratio. But the ejector could operate at a higher critical back pressure and would therefore be particularly suitable for changing condenser conditions in real applications.

### 3.2. Effect of Range of the Length of the Constant-Area Mixing Section

**Figure 7:**
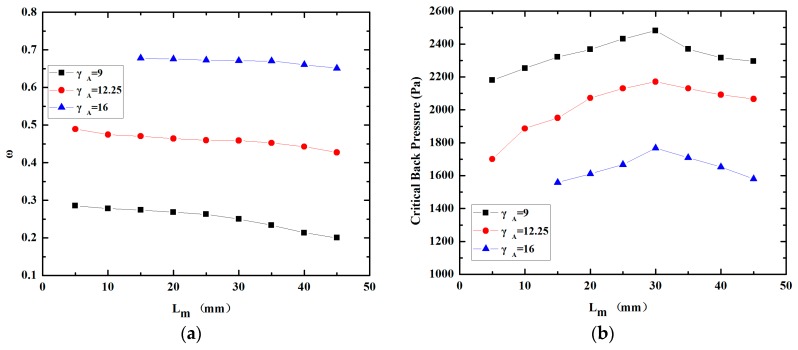
Variation of performance with the length of constant-area mixing section; (**a**) Variation of entrainment ratio; (**b**) Variation of critical back pressure.

The effect of the length of the constant-area mixing section, L_m_, on the performance of the miniature ejector is shown in Figure 7. The length of the constant-area mixing section varied in the range of 5 mm–45 mm with an increment of 5 mm. Figure 7a illustrates the variation of the entrainment ratio with the increase of the length of constant-area mixing section. It can be seen from the curves that the entrainment ratio declines with increasing L_m_. To be specific, decline of the entrainment ratios for γ_A_ = 9, γ_A_ = 12.25 and γ_A_ = 16 are 0.08, 0.05 and 0.03 when L_m_ varies from 5 mm to 45 mm, respectively. Also, the tendency shown in Figure 7a reveals clearly that the effect of $L_{m}$ on the entrainment ratio becomes smaller when γ_A_ increases. Figure 7b indicates that the critical back pressure rises initially and then decreases with increasing L_m_. The peak values of the critical back pressure for γ_A_ = 9, γ_A_ = 12.25 and γ_A_ = 16 are 2480 Pa, 2142 Pa and 1761 Pa at the L_m_ = 30 mm, respectively. It is worth noting that the critical mode region can’t be found when γ_A_ = 16 and L_m_ = 5 mm or 10 mm, which means the ejector can’t function. What’s more, the critical back pressures are all lower than the saturation pressure of water vapor at 15 °C, when γ_A_ = 12.25, L_m_ = 5 mm and γ_A_ = 16, L_m_ =5 mm–45 mm. In other words, the structures stated above are not recommended for applying in practice.

**Figure 8:**
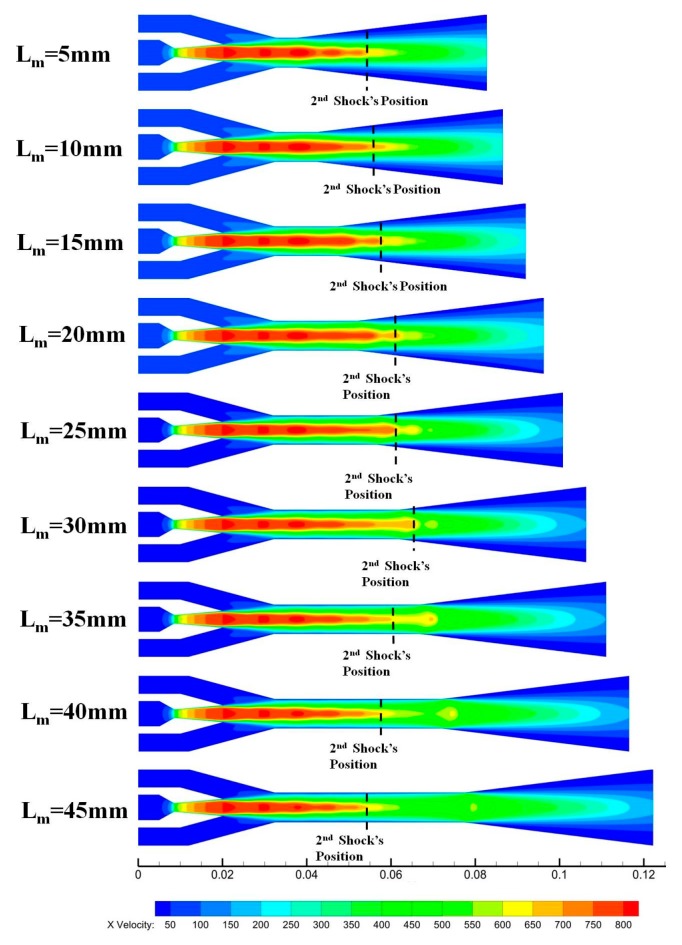
Velocity contours at different length of the constant-area mixing section.

To further study flow phenomena inside the ejector, velocity contours at different lengths of the constant-area mixing section of γ_A_ equals 12.25 are shown in Figure 8. It is easy to see that the profiles of the ‘*wing-shaped*’ area in the mixing chambers were similar to each other when L_m_ varied from 5mm to 45 mm, and the ‘*wing-shaped*’ area decreases with increasing L_m_. This corresponds with the variation of the entertainment ratio shown in Figure 7a. In addition, the variation trend of the critical back pressure is in keeping with the position of the second shock shown in Figure 8. And the second shock position has been marked by dash in Figure 8. The 2nd shock position of the ejector with L_m_ = 30 mm is the farthest one away from the nozzle when γ_A_ = 12.25. The mixed flow has more momentum at the exit of the diffuser, which can gain a higher critical back pressure. As a result, L_m_ = 30 mm is the optimal length of the constant-area mixing section for primary flow and second flow to mix when γ_A_ = 12.25.

### 3.3. Effect of Nozzle Exit Position

**Figure 9:**
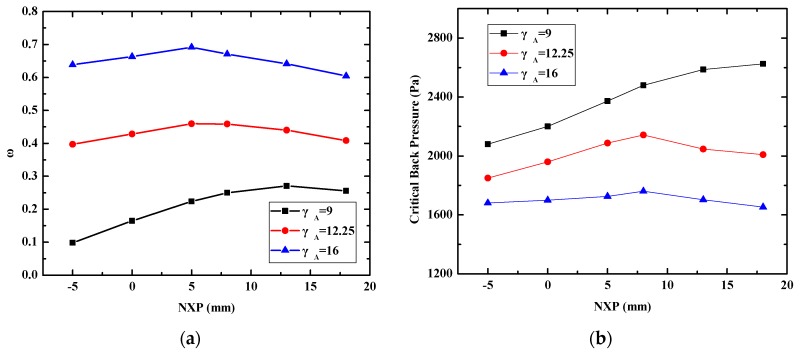
Variation of the performance with different nozzle exit position (NXP); (**a**) Variation of entrainment ratio; (**b**) Variation of critical back pressure.

The nozzle exit position is another very important parameter. The effect of the nozzle exit position on the performance of the ejector with different γ_A_ is shown in Figure 9. NXP varied in the range of −5 mm to 18 mm. The variation trend of the entrainment ratio with different NXPs is illustrated in Figure 9a. It is found that the three curves go up initially and then decline with increasing NXP. There is an optimal NXP for each ejector. More precisely, the optimal NXPs of the entrainment ratio are 13 mm, 8 mm and 5 mm for γ_A_ = 9, 12.25 and 16 respectively. Correspondingly, the peak values of the entrainment ratio are 0.27, 0.46 and 0.69. The variation trend of the critical back pressure with different NXP is shown in Figure 9b. It can be found that the three curves have different trends. When γ_A_ = 9, the critical back pressure increases with increasing the NXP. When γ_A_ = 12.25, the critical back pressure increases first and then decreases with increasing the NXP. It is a coincidence that the optimal NXP of the critical back pressure is 8 mm, which is as the same as that of the entrainment ratio. When γ_A_ = 16, the critical back pressure has no evident changes in the range of NXP = −5 mm to 18 mm. It shows that the critical back pressure is not sensitive to NXP variations. The factors contributing to this phenomenon are complex. We thought the cause might be “*entrained duct*” formed by the mixing chamber and nozzle outlet has an optimum “*effective area*” at different NXPs.

## 4. Conclusions

In this study, CFD is employed to investigate the effects of the area ratio of the ejector constant-area mixing section to nozzle throat, the length of constant-area section, and the NXP, on the performance in a miniature steam ejector embedded with a capillary pump loop. According to the results presented, it can be concluded that the performance of the miniature steam ejector is very sensitive to the area ratio of the constant-area mixing section to the nozzle. For the needs of practical application, the area ratio should be smaller than 16. In addition, the length of the constant-area mixing section has more effect on the critical back pressure than the entrainment ratio. The position of the second shock reflects that the total momentum of the mixed fluid and the critical back pressure increase when the position moves toward the diffuser. The miniature steam ejector will operate at higher critical back pressure when the second shock position moves toward the diffuser. There is an optimal length of constant-area section, L_m_ = 30 mm, where the critical back pressure has a peak value for the three ejectors with given area ratios. Furthermore, the effects of the NXP on the entrainment ratio and the critical back pressure are different for the three ejectors with given area ratios. The results in this paper could be used to optimize the geometry parameters of the miniature ejector before fabricating a real prototype.

## Figures and Tables

**Table 1:** Geometry parameters of the ejector.

Parameter	Value	Units
Nozzle throat diameter $D_{n}$	2	mm
Nozzle exit diameter $D_{a}$	4	mm
Nozzle convergent angle $\theta_{1}$	17	°
Nozzle divergent angle $\theta_{2}$	10	°
Nozzle exit position	−5, 0, 5, 8, 13, 18	mm
Length of constant-area mixing section $L_{m}$	5, 10, 15, 20, 25, 30, 35, 40, 45	mm
Diameter of constant-area mixing section $D_{m}$	6, 7, 8	mm
Mixing chamber Convergence angle $\theta_{3}$	15	°
Diffuser Exit diameter $D_{o}$	18	mm
Diffuser Divergent angle $\theta_{4}$	7	°
